# Avoiding Misdiagnosis and Missed Diagnosis for Appropriately Treating Spinal Osteoid Osteomas: A Single‐Center Experience

**DOI:** 10.1111/os.13280

**Published:** 2022-04-18

**Authors:** Qiming Xu, Wensheng Liu, Hairong Xu, Lijia Cui, Yuan Li, Huachao Shan, Zhen Huang, Ke Ma, Xiaohui Niu

**Affiliations:** ^1^ Department of Orthopaedic Oncology Beijing Jishuitan Hospital Beijing China; ^2^ Department of Endocrinology Peking Union Medical College Hospital, Chinese Academy of Medical Sciences and Peking Union Medical College, Key Laboratory of Endocrinology, National Health Commission Beijing China

**Keywords:** Misdiagnosis, Missed diagnosis, Osteoid osteoma, Spine, Surgery

## Abstract

**Objectives:**

To analyze the causes of misdiagnosis and missed diagnosis in spinal osteoid osteoma, and to put forward solutions to improve diagnosis accuracy and treatment efficacy in patients.

**Methods:**

We performed a retrospective cohort study on patients with spinal osteoid osteoma in Beijing Jishuitan Hospital from January 1983 to September 2019. All patients underwent surgery. The outcome measures were the extent of local pain, nocturnal pain, radicular symptoms of extremities after surgery, and reduction or disappearance of lesions on CT after surgery.

**Results:**

Thirty‐seven patients with spinal osteoid osteoma were recruited in the study. A total of 27% were female, and the mean (SD) age at diagnosis was 21.3 (8.7) years. A total of 87.0% of patients presented with nocturnal pain, and 94.7% of patients were responsive to NSAIDS treatment. The mean (SD) time from the initial onset of symptoms to the final diagnosis was 14.7 (12.5) months. Only four of 37 (10.8%) patients were correctly diagnosed with spinal osteoid osteoma on the first visit to the local hospital. CT is associated with a higher diagnosis rate than X‐ray or MRI on the first visit. Surgical navigation was used in 88.9% of patients who underwent curettage resection, and in 10% of patients who underwent en bloc resection. A total of 37 of 37 patients (100%) reported relief of local pain and radicular symptoms of extremities after surgery, and no recurrence of tumors was found during follow‐ups.

**Conclusions:**

Spinal CTs are recommended to be performed if osteoid osteoma is suspected based on clinical manifestation, including nocturnal pain and responsiveness to NSAIDS treatment, to avoid misdiagnosis and missed diagnosis of spinal osteoid osteoma.

## Graphical abstract

In a cohort of spinal osteoid osteoma patients, most of them had typical nocturnal pain and were responsive to NSAIDS, which were often neglected and resulted in misdiagnosis or missed diagnosis. X‐ray showed no specific signs or only scoliosis, MRI showed an obscure signal of the nidus, whereas CT scan revealed a well‐defined nidus with strong contrast with adjacent reactive bone. We recommend patients with typical manifestations above to have CT performed to confirm spinal osteoid osteoma.

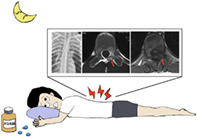

## Introduction

Osteoid osteoma accounts for about 10% of benign bone tumors^1^. Most osteoid osteomas occur between the ages of 20 to 30 years, with a male to female ratio of 2:1‐3:1^2^. About 10% of osteoid osteoma occurs in the spine^3^, mostly involving the posterior elements of the spine^4^. Osteoid osteoma is pathologically characterized by a nidus of un‐mineralized immature osteoid tissue surrounded by varying degrees of reactive sclerotic bone^5^. The nidus of osteoid osteoma is always less than 1.5 cm in diameter, which easily leads to neglection in radiological examination and missed diagnosis or misdiagnosis^6^.

X‐rays of osteoid osteoma in long bones typically manifests a small lucent spot, surrounded by increased bone formation^7^. However, osteoid osteoma in the spine always shows no specific signs in X‐rays due to the complicated anatomical structure of the vertebra and the disturbance by neighboring organs and tissues. Although the tumor nidus of spinal osteoid osteoma and the reactive sclerotic bone around contrast sharply in CT scans^5^, the lesion is still likely to be neglected due to the tininess of the tumor.

Benign as it is, spinal osteoid osteoma impairs physical activity, causes nocturnal pain and radicular symptoms of extremities, and severely lowers quality of life in patients. Although osteoid osteomas may remit in over 2 to 10 years, misdiagnosis and missed diagnosis of them can lead to delayed^5^, and more seriously, inappropriate treatment, which brings short‐term or long‐term harm to patients. This includes unbearable pain, abuse of analgesics, and structural spinal scoliosis. Therefore, it is urgent to improve the diagnosis accuracy in spinal osteoid osteoma. Once the diagnosis of osteoid osteoma is confirmed, the need for surgical intervention is indicated by whether the patient has pain that is unable to be controlled by medication or the tendency to develop a structural scoliosis^8^

.Current research in spinal osteoid osteoma mainly focuses on various surgical interventions. The traditional surgical approaches for spinal osteoid osteoma include curettage and en bloc excision^9^, ^10^. Recently, minimally invasive interventions including CT‐guided excision and radio‐frequency ablation have also been encouraged^3^, ^11^, ^12^. However, there have limited studies on systemically analyzing the factors contributing to misdiagnosis and missed diagnosis of spinal osteoid osteoma or presenting strategies to eliminate misdiagnosis and missed diagnosis of the disease.

We performed a retrospective cohort study on spinal osteoid osteoma patients in the Beijing Jishuitan Hospital. The aims of this study are: (i) to analyze the causes of misdiagnosis and missed diagnosis in spinal osteoid osteoma; (ii) to put forward solutions to improve diagnosis accuracy and treatment efficacy in patients.

## Methods

### Inclusion and Exclusion Criteria

The inclusion criteria of the study were as follows: (i) patients with local neck or back pain and/or radicular symptoms of extremities, who visited the Department of Orthopedic Oncology of the Beijing Jishuitan Hospital from January 1983 to September 2019; (ii) pathological diagnosis was spinal osteoid osteoma; (iii) exposure factor: patients who underwent surgery on the spinal lesion. The exclusion criteria included: (i) incomplete medical records; (ii) loss of follow‐up.

The outcome measures included Visual Analog Scale (VAS) of local pain and nocturnal pain after surgery, extent of radicular symptoms of extremities after surgery, and reduction or disappearance of lesions on CT after surgery.

### Standardized Description of Indicators

**Local pain**: local pain refers to localized neck or back pain with VAS above 3, which is considered as moderate to worst pain. VAS for pain is on a scale of a 10‐cm horizontal line used to determine the pain intensity, self‐accounted by the patients, with the 0‐cm mark referring to no pain and the 10‐cm mark referring to worst pain^13^.

**Radicular symptoms of extremities**: radicular symptom of extremities refers to conditions involving inflammatory irritation or mechanical compression of the spinal nerve root at the level of the lesion. Radicular symptoms include radicular pain, muscle weakness, parenthesis, and claudication.

**Nocturnal pain**: nocturnal pain refers to local or extremity pain which affects patients' sleep quality during nighttime.

**Responsive to NSAIDS treatment**: patients responsive to non‐steroidal anti‐inflammatory drugs (NSAIDS) treatment refers to those who have significant relief of local pain or radicular symptoms of extremities with NSAIDS treatment.

### Radiological Measurement and Localization

Patients underwent CT, MRI, and X‐ray before and after surgery. The tumor size was described by measuring the anterior–posterior diameter, left–right diameter, cephalad‐caudal diameter in CT. The tumor location was described using Weinstein‐Boriani‐Biagini (WBB) sector in CT. WBB sector is a surgical staging system for describing the anatomical location of spinal tumors^14^. The vertebra is divided clockwise into 12 radiating zones. Zone 1 and 12 refer to the left and right half of the spinous process, Zone 2 and 11 refer to the left and right superior articular facet, Zone 3 and 10 refer to the left and right transverse process, Zone 4 and 9 refer to the left and right pedicle, and Zone 5–8 refer to the vertebral body.

### Statistical Analysis

Descriptive statistics were performed using mean/standard deviation or frequency/percentage, as appropriate. χ^2^ tests or *t*‐tests were performed as appropriate to compare between‐group variance in R (version 4.0.0, R Foundation for Statistical Computing, Vienna, Austria). We chose a statistically significance cutoff of *P* value ≤0.05 for two‐sided tests.

## Results

### Demographic Features of the Patients

Thirty‐eight patients with spinal osteoid osteoma who underwent surgery were recruited, among whom one was excluded, as the medical record was incomplete (Table 1). The median (range) follow‐up period is 7.9 (1.9–38.6) years. Ten of 37 patients (27%) were female. The mean (SD) age at diagnosis was 21.3 (8.7) years. All patients presented local pain. Twenty of 37 patients (54.1%) had radicular symptoms of extremities, 20 of 23 patients (87.0%) presented nocturnal pain, and 18 of 19 patients (94.7%) were responsive to NSAIDS treatment. Three of 37 patients (8.1%) had osteoid osteoma in the vertebral body, and 34 of 37 patients (91.9%) had nidus in the vertebral appendix. The mean (SD) diameter of osteoid osteoma was 1.1 (0.9) cm (anterior–posterior diameter), 1.2 (1.1) cm (left–right diameter) and 1.1 (0.9) cm (cephalad‐caudal diameter), respectively (Table 1).

**TABLE 1:** Demographic characteristics of the patients with spinal osteoid osteoma

Demographics	*n* (%) or mean ± standard deviation
Female	10 (27.0%)
Age at diagnosis (years)	21.3 ± 8.7
Time from initial symptom to first visit (months)	5.8 ± 7.4
Time from initial symptom to final diagnosis (months)	14.7 ± 12.5
Symptom	
Local pain	37 (100%)
Radicular symptom of extremities*	20 (54.1%)
Nocturnal pain	20 (87.0%)
Responsive to NSAIDS treatment	18 (94.7%)
High uptake in whole‐body bone scintigraphy	15 (100%)
Spinal location	
Cervical	8 (21.6%)
Thoracic	9 (24.3%)
Lumbar	16 (43.2%)
Sacral	4 (10.8%)
Tumor size	
Anterior–posterior diameter (cm)	1.1 ± 0.9
Left–right diameter (cm)	1.2 ± 1.1
Cephalad‐caudal diameter (cm)	1.1 ± 0.9
Tumor location (WBB sectors^†^)	
2–4	12 (32.4%)
9–11	14 (37.8%)
12–1	4 (10.8%)
5–8	3 (8.1%)
Tumor location (anatomical location)	
Vertebral body	3 (8.1%)
Vertebral appendix	34 (91.9%)

* radicular symptom of extremities include: radicular pain, muscle weakness, paresthesia, muscle spasm, claudication.

^†^ WBB: Weinstein – Boriani – Biagini.

### Misdiagnosis and Missed Diagnosis at Local Hospitals

Four of 37 patients (10.8%) were correctly diagnosed with osteoid osteoma on the first visit to local hospital (Table 2). The mean (SD) time from initial symptoms to final confirmed diagnosis was 14.7 (12.5) months (Table 1). The most common misdiagnosis was lumbar muscle degeneration (Table 2). Only one patient was misdiagnosed with intervertebral disc herniation on the first visit, although there were 20 patients with radicular symptoms of extremities. Three patients were misdiagnosed with diseases of other systems (Table 2).

**TABLE 2:** Summary of diagnosis of patients with spinal osteoid osteoma patients at first visit in local hospital

Diagnosis at first visit	*n* (%)
Spinal osteoid osteoma	4 (10.8%)
Misdiagnosis as other spinal disease	
Lumbar muscle degeneration	7 (18.9%)
Intervertebral disc herniation	1 (2.7%)
Spinal scoliosis	1 (2.7%)
Spinal tuberculosis	1 (2.7%)
Spinal angioma	1 (2.7%)
Uncategorized spinal tumor	1 (2.7%)
Undifferentiated spondyloarthropathy	1 (2.7%)
Back pain with unknown reason	8 (21.6%)
Misdiagnosis as diseases of other systems	
Appendicitis*	1 (2.7%)
Lung tuberculosis^†^	1 (2.7%)
Multiple sclerosis^‡^	1 (2.7%)
Not available	9 (24.3%)

* One patient had a thoracic osteoid osteoma which impinged into the spinal canal, leading to myelopathic symptoms, and was thus misdiagnosed with multiple sclerosis at the local hospital and wrongly treated with high doses of glucocorticoid.

^†^ One patient had osteoid osteoma on the transverse process of thoracic spine, which mimicked nodules on the hilus of the lung in X‐ray and was misdiagnosed with lung tuberculosis at the local hospital and wrongly treated with anti‐tuberculosis therapy.

^‡^ One patient had osteoid osteoma in the right part of the fifth lumbar vertebral body, presented referred pain of right lower quadrant of the abdomen, and was misdiagnosed with appendicitis at local hospital and had a wrong surgery of appendicectomy.

### Radiographic Examination

**Figure 1:**
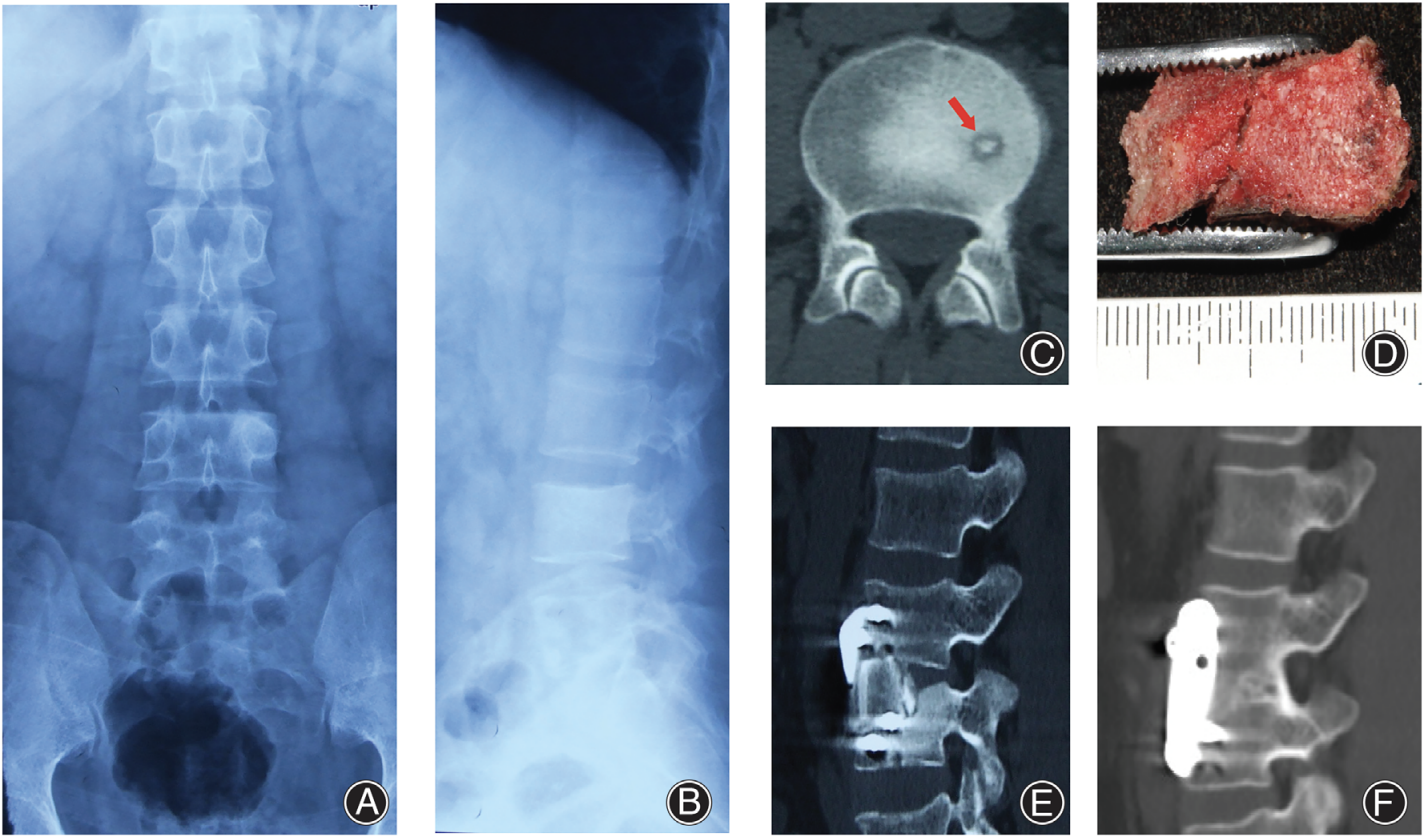
A 29‐year‐old male patient with lumbar spinal osteoid osteoma. Anterior–posterior (A) and lateral (B) lumbar spine X‐ray was performed and revealed no specific signs, and the patient was misdiagnosed with lumbar muscle degeneration at the first visit in the local hospital. (C) CT revealed a well‐defined nidus with strong contrast with the adjacent reactive bone located in the lumbar spine L_4_. (D) En bloc resection was performed, and specimen was pathologically diagnosed with osteoid osteoma. (E) After resection of the tumor, intervertebral fusion and fixation was performed with autograft bone of anterior superior iliac spine and anterior screw‐plate system, which was showed in the immediate post‐operative CT. (F) In the last follow‐up 12 years after the surgery, CT revealed successful intervertebral fusion and no recurrence of tumor.

**Figure 2:**
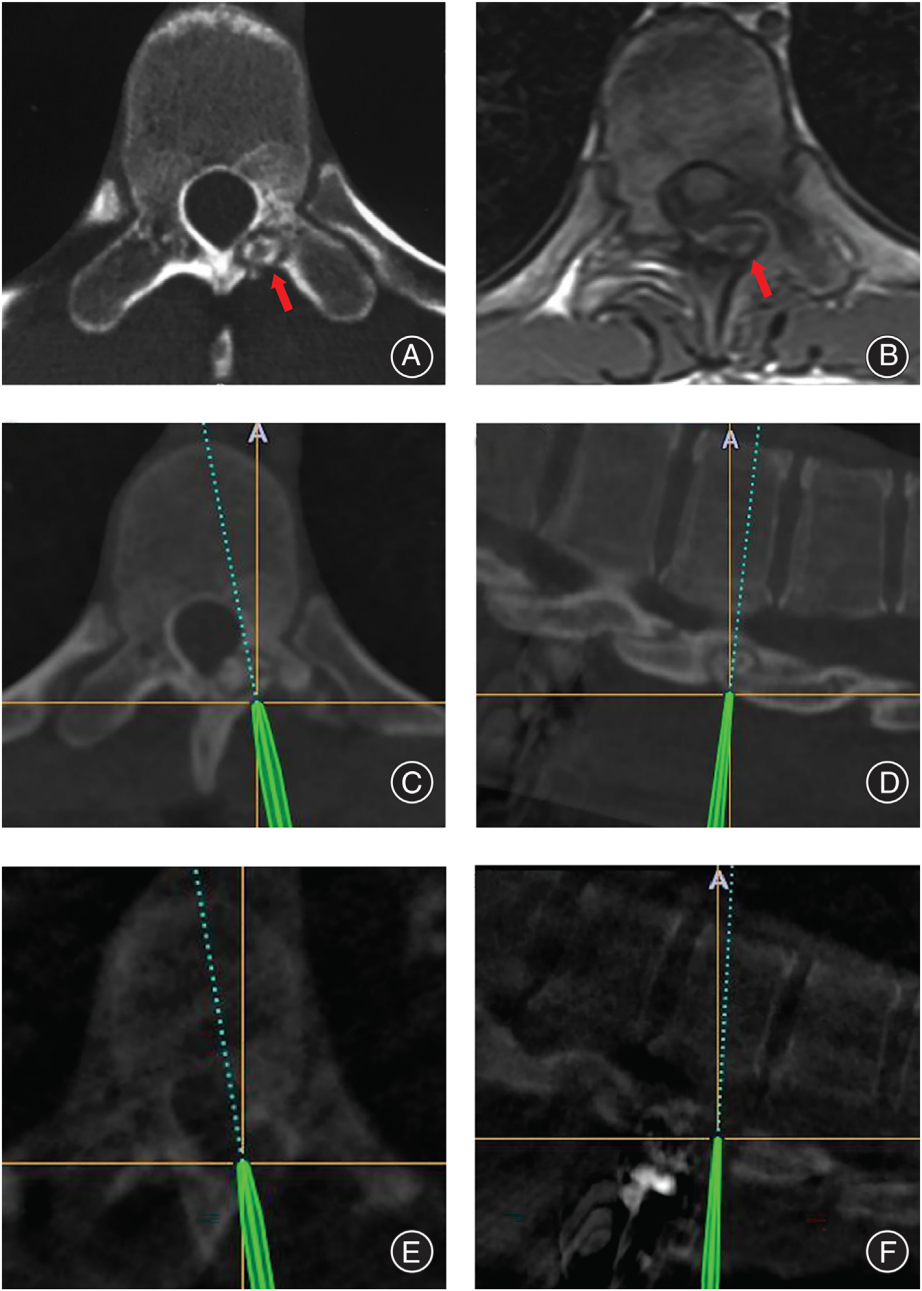
A 30‐year‐old male patient with thoracic spinal osteoid osteoma. (A) CT revealed a well‐defined nidus located in the vertebral laminae of the thoracic spine T_12_. (B) In the same segment of MRI, the signal of the nidus was obscure with no strong contrast with surrounding bone. (C, D) Computer‐assisted navigation was used in the surgery, to obtain precise location of the nidus simultaneously in three‐dimensions. (E, F) After curettage of the nidus, intraoperative CT was performed to confirm the tumor was completely removed.

The nidus of spinal osteoid osteoma appears to be well‐defined in CT (Figures 1C and 2A). In MRI, however, the signal of nidus is always obscure with no strong contrast with the surrounding reactive bone (Figure 2B). In X‐ray, osteoid osteoma always shows no specific signs (Figure 1A and B) and leads to a missed diagnosis.

The radiographic examination on the first visit is significantly associated with the misdiagnosis rate (*P* = 0.0005) (Supplementary Table 1). In those eight patients who underwent spinal CT on the first visit, four of them (50%) were correctly diagnosed with osteoid osteoma. However, in the 27 patients who underwent either X‐ray or MRI on the first visit, the misdiagnosis rate was 100%. Six of 37 patients (16.2%) had core needle aspiration biopsy performed before surgery, but the pathological sections showed only inflammatory cells, without tumor cells.

### Surgery Procedure and Surgery Approach

Regarding surgical procedure, en bloc resection (Figure 1D, E and F) was performed in 10 (27.0%) patients, and curettage resection (Figure 2C, D, E and F) was performed in 27 (73.0%) patients (Table 3). The use of intraoperative computer‐assisted navigation is significantly associated with the selection of surgical procedures. Computer‐assisted navigation (Figure 2C, D, E and F) was used in 24 of 27 patients (88.9%) who underwent curettage resection, and one of 10 patients (10%) who underwent en bloc resection (*χ* ^*2*^ = 17.28, *P* < 0.0001) (Table 3). The use of fixation and bone graft was not significantly different in the en bloc group and curettage group.

**TABLE 3:** Summary of surgical procedure

	En bloc (*n* = 10)	Curettage (*n* = 27)	χ^2^ value*	*P* value^†^
Use of intraoperative computer‐assisted navigation	1 (10%)	24 (88.9%)	17.28	<0.0001
Use of fixation	2 (20%)	2 (7.4%)	0.2494	0.6175
Use of bone graft	1 (10%)	2 (7.4%)	<0.001	1

* χ^2^ test was performed.

^†^ *P* ≤ 0.05 was considered as statistically significant.

Tumor locations associated with the selection of surgery approaches. Anterior approach was used significantly more when the WBB sectors of the tumor were 5–8 (i.e. located in the anterior part of the vertebrae), and posterior approach was used significantly more when the WBB sectors of the tumor was 2–4/9–11 (i.e. located in the lateral part of the vertebrae) or 12–1 (i.e. located in the posterior part of the vertebrae) (*χ* ^*2*^ = 13.299, *P* = 0.0013) (Supplementary Table 2).

### Prognosis

All 37 patients (100%) with osteoid osteoma reported relief of local pain and radicular symptoms of extremities after surgery, regardless of the surgical approach. All patients had no recurrences, both symptomatically and radiologically, during the follow‐ups.

Scoliosis was found in 27 of 37 patients (73%) before surgery, which may be associated with the symptom of back pain. Six of 27 patients (22.2%) had scoliosis corrected after surgery, and 21 of 27 patients (77.8%) remained with scoliosis after surgery (Supplementary Table 3). The scoliosis correction rate was not associated with the age at diagnosis (*t*  = 0.1883, *P* = 0.8557) nor the time from initial symptoms to final diagnosis (*t*  = −1.7078, *P* = 0.1016).

## Discussion

To our knowledge, this is the largest cohort study focusing on the misdiagnosis and missed diagnosis of spinal osteoid osteoma. We found that misdiagnoses and missed diagnoses were extremely common in spinal osteoid osteoma, as only 10.8% of patients in this study were correctly diagnosed with osteoid osteoma on the first visit to the local hospital. Misdiagnoses or missed diagnoses of spinal osteoid osteoma were only present in several case reports previously^15^, ^16^. We also found that characteristic clinical manifestations in spinal osteoid osteoma patients included nocturnal pain and responsiveness to NSAIDS treatment, and CT was associated with a higher diagnosis rate of spinal osteoid osteoma than X‐ray or MRI. These findings would improve the diagnosis accuracy in spinal osteoid osteoma, and were accordant with previous reports^17^, ^18^. Besides, we found that all patients with spinal osteoid osteoma reported relief of local pain and radicular symptoms of extremities after surgery, and no recurrence of tumors was found during follow‐ups. Previous studies reported that the recurrent rate of osteoid osteoma of en bloc surgery or curettage surgery varied from 4.5%–12%^19^, ^20^. These findings indicated that once the diagnosis of spinal osteoid osteoma was made, prompt surgery had favorable prognosis.

### Causes of Misdiagnosis and Missed Diagnosis of Spinal Osteoid Osteoma

In this study, we found that spinal osteoid osteoma commonly mimicked other spinal diseases, including lumbar muscle degeneration, scoliosis, and intervertebral disc herniation. Firstly, lumbar muscle degeneration was the most common misdiagnosis. We speculated that since no obvious lesion was found in the MRI or X‐ray in some patients, the symptom of spinal pain was wrongly attributed to lumbar muscle degeneration on the first visit. Besides, scoliosis was found in 73.0% of patients with spinal osteoid osteoma in this study. Previous studies also reported two cases of spinal osteoid osteoma that were misdiagnosed as scoliosis^21^, ^22^. “Painful” scoliosis could be due to the asymmetric muscle spasm, and the nidus was typically on the concave side of the lumbar curve. Intervertebral disc herniation was another misdiagnosis of spinal osteoid osteoma, which can present with back pain and sciatica as initial symptoms. Although there were few cases of osteoid osteoma misdiagnosed as intervertebral disc herniation both in our cohort and in previous reports^15^, it should be taken into consideration especially when patients experience long duration of back pain and sciatica.

In addition to the spinal diseases above, spinal osteoid osteoma was also found to mimic diseases of other systems, and misdiagnoses led to serious consequences. In this cohort, three patients with spinal osteoid osteoma were misdiagnosed with multiple sclerosis, lung tuberculosis and appendicitis at local hospitals, and were all given wrong treatments (Table 2). Although those misdiagnoses seemed inconceivable, the particular characteristics of each case tempted the doctors to make a incorrect diagnosis, which could have been avoided if characteristic clinical manifestations were inquired about in detail and spinal CT scans were taken with circumspective investigation.

### How to Improve Diagnosis Accuracy of Spinal Osteoid Osteoma

Firstly, detailed inquiry of nocturnal pain and responsiveness to NSAIDS is the key point to improvement of diagnosis accuracy of spinal osteoid osteoma. We found that 87.0% of patients presented with nocturnal pain, and 94.7% of patients were responsive to NSAIDS treatment (Table 1). Extremely high levels of prostaglandins have been reported in osteoid osteomas^18^, ^23^, which are considered to be responsible for pain generation. NSAIDS can reduce prostaglandins synthesis and therefore relieve pain caused by osteoid osteoma. The response rate to NSAIDS of osteoid osteoma varies in different studies^17^. Pettine *et al*. reported a response rate of 90% to NSAIDS^24^, which is similar to our data. Thus, we recommend that clinicians should carefully collect medical history of nocturnal pain and responsiveness to NSAIDS treatment in patients with spinal disease.

Secondly, spinal CT scans with circumspective investigation is essential to making an accurate diagnosis of spinal osteoid osteoma. We found that there is always a high contrast between the tumor nidus of osteoid osteoma (in low density) and the surrounding reactive sclerotic bone (in high density) in CT (Figures 1C and 2A), which should be differentiated with other tumors or tumor‐like lesions with similar characteristics, including osteoblastoma and intracortical abscess. Osteoblastoma is characterized by a nidus with diameter over 2 cm^25^, while intracortical abscess is characterized by inflammatory symptoms and irregular pain rather than regular nocturnal pain in osteoid osteoma^26^. Other researchers also reported that the detection rate of spinal osteoid osteomas by CT is almost 100%^18^, ^27^. However, in MRI, the tumor nidus and surrounding reactive bone presented similar signals (Figure 2B), which led to difficulty in diagnosis^28^. In X‐ray, the tiny osteoid osteoma is often shielded by the cortical bone of the vertebral appendix, which leads to missed diagnosis. In our cohort, four of eight patients who underwent spinal CT on the first visit were correctly diagnosed with osteoid osteoma. However, zero of 27 patients who underwent either X‐ray or MRI on the first visit were correctly diagnosed on the first visit. Assoun *et al*. compared the diagnostic accuracy of CT and MRI in 19 patients with osteoid osteoma before surgery and found that CT was more accurate than MRI in the detection of osteoid osteoma nidus in 63% of cases^29^ . Thus, if osteoid osteoma is suspected based on clinical manifestations, we recommend spinal CT rather than X‐ray or MRI to be prescribed to detect the lesion.

Thirdly, although the definite diagnosis of spinal osteoid osteoma is dependent on pathological findings, we found that aspiration biopsy would not improve diagnosis accuracy in spinal osteoid osteoma. Six patients in this cohort had performed core needle aspiration biopsy before surgery, but pathological sections showed only inflammatory cells, without tumor cells. Soliman *et al*. reported that in 42 patients with osteoid osteoma who underwent intraprocedural biopsy immediately prior to radiofrequency ablation, only 52.3% of the biopsies were adequate for histological diagnosis of osteoid osteoma, even with the guidance of thinner intraprocedural CT^30^. In our study, the median diameter of osteoid osteomas was only 1.1–1.2 cm (Table 1). Thus, aspiration biopsy may miss the nidus of the tumor and only acquire nonspecific inflammatory tissues in the reactive bone. Therefore, we do not recommend aspiration biopsy for spinal osteoid osteoma, as a false negative result may occur and mislead clinicians. The definite diagnosis of spinal osteoid osteoma should be confirmed by the pathology of specimen obtained from resection operations.

### How to Improve Treatment Efficacy of Spinal Osteoid Osteoma

Firstly, traditional open surgeries, including en bloc resection or curettage, were considered as the standard therapy for spinal osteoid osteoma to improve treatment efficacy, which is also advocated by our center. Recently, minimally invasive techniques, including CT‐guided percutaneous excision, radiofrequency ablation, laser ablation, and cry ablation, have been used in other centers with reported advantages of lower cost and lower invasiveness, especially in osteoid osteomas with difficult‐to‐access locations in the spine^3^, ^31^. However, the short‐term and long‐term success rates of those minimally invasive techniques are uncertain^32^, and neurological complications are especially concerning. In our cohort, all patients underwent open surgery. It is urgent for future studies to compare the treatment effects and complications between open surgery and minimally invasive techniques in treating spinal osteoid osteoma.

Secondly, computer‐assisted navigation is helpful to improving treatment efficacy in both en bloc resection and curettage of spinal osteoid osteoma. In our cohort, regardless of the surgical approach of open en bloc resection or curettage, 100% of patients reported relief of local pain and radicular symptoms of extremities after surgery, and no recurrence of tumors was found during follow‐up of more than 12 months. Healey *et al*. reported that en bloc resection of osteoid osteoma had the lowest recurrence rate, while intralesional resection or curettage had the highest recurrence rate^20^. Sluga *et al*. reported that relapse was less frequent after en bloc excision than curettage (4.5% *vs* 12%)^19^. We attributed the difference between our cohort and previous studies to the use of computer‐assisted surgical navigation. In our study, computer‐assisted navigation was used in 88.9% of patients who underwent curettage resection, and 10% of patients who underwent en bloc resection. Computer‐assisted navigation allows for more precise resections of the tumor on a smaller scale, thus preserving the stability and function of the spine, and avoiding residuals of the tiny tumor. Thus, we recommend surgical navigation to be used for precise resection of the spinal osteoid osteoma.

### Limitations

There are certain limitations in this study. First, this is a single‐center study, which limited our comparison of open surgery with other minimally invasive techniques in treating spinal osteoid osteoma. Second, this retrospective study had a large time span, and the diagnosis experience and imaging techniques varied at different times. In the future, multi‐center prospective studies are needed to further investigate the optimal diagnosis and treatment strategies for spinal osteoid osteoma.

## Conclusion

Misdiagnosis and missed diagnosis are extremely common in patients with spinal osteoid osteoma. We recommend patients to have spinal CTs performed if osteoid osteoma is suspected based on clinical manifestation, including nocturnal pain and responsiveness to NSAIDS treatment. Once the diagnosis of spinal osteoid osteoma is confirmed, surgery with aid of computer‐assisted navigation is the first choice of treatment as it provides satisfactory outcomes.

## Conflict of Interest

Authors declare that no conflict of interest exists in the study.

## Supporting information


**Appendix S1.** Supporting information.
