# Supplementary material for: Avoiding Misdiagnosis and Missed Diagnosis for Appropriately Treating Spinal Osteoid Osteomas: A Single‐Center Experience
Source: Orthop Surg. 2022 Apr 18;14(5):868–75. doi: 10.1111/os.13280 (PMC9087446; doi:10.1111/os.13280)
Supplement: Supplementary file 1 — Appendix S1. Supporting information. [file OS-14-868-s001.docx]

**Supplementary material.**

**Supplementary Table 1. Summary of radiographic examination and misdiagnosis rate on the first visit in patients with spinal osteoid osteoma.**

| Radiographic examination on the first visit | Misdiagnosis cases on the first visit (%) | χ^2^ value ^†^ | *p* value^‡^ |
| --- | --- | --- | --- |
| Spinal X-ray (N = 15) | 15 (100%) | 15.242 | 0.0005 |
| Spincal MRI (N = 12) | 12 (100%) |  |  |
| Spinal CT (N = 8) | 4 (50%) |  |  |

^†^ χ^2^ test was performed.

^‡^ *p*≤0.05 was considered as statistically significant.

**Supplementary Table 2. Summary of surgery approach and tumor location.**

| WBB sectors^†^ (the location of spinal tumor) (N) | Anterior approach (N) | Posterior approach (N) | χ^2^ value^‡^ | *p* value^*^ |
| --- | --- | --- | --- | --- |
| 2-4/9-11(lateral) (N=26) | 1 | 25 | 13.299 | 0.0013 |
| 12-1 (posterior) (N=4) | 0 | 4 |  |  |
| 5-8 (anterior) (N=3) | 2 | 1 |  |  |

^†^ WBB: Weinstein – Boriani – Biagini.

^‡^ χ^2^ test was performed.

^*^ *p*≤0.05 was considered as statistically significant.

**Supplementary Table 3. Summary of scoliosis correction rate after surgery.**

|  | Scoliosis corrected after surgery (n = 6) | Scoliosis not corrected after surgery (n = 21) | *t* value^†^ | *p* value^‡^ |
| --- | --- | --- | --- | --- |
| Age at diagnosis | 20.8±9.2 | 20.0±8.2 | 0.1883 | 0.8557 |
| Time from initial symptom to final diagnosis | 9.2±5.9 | 16.3±15.5 | -1.7078 | 0.1016 |

^†^ t-test was performed

^‡^ *p*≤0.05 was considered as statistically significant.
